# Impact of the influx of Syrian refugees on domestic violence against Jordanian women: Evidence from the 2017–18 Jordan Population and Family Health Survey

**DOI:** 10.1371/journal.pone.0288144

**Published:** 2023-11-08

**Authors:** Han Choi, Sebastian Bauhoff

**Affiliations:** Department of Global Health and Population, Harvard T.H. Chan School of Public Health, Boston, Massachusetts, United States of America; Tokyo Medical and Dental University: Tokyo Ika Shika Daigaku, JAPAN

## Abstract

The 2011 Syrian crisis led to a large influx of refugees into neighboring countries, including Jordan. The resulting stress on local host communities could heighten the risk of domestic violence against Jordanian women. We utilized multilevel propensity score weighting and data from the 2017–18 Jordan Population and Family Health Survey to empirically test for differences in outcomes related to domestic violence, marital control, and justification of wife-beating between Jordanian communities with varying density levels of Syrian women. We did not find systematic differences in these outcomes across communities. However, we cannot rule out effects that may not be statistically detectable with our sample but could still be substantively meaningful.

## Introduction

Globally, more than 700 million women have experienced physical or sexual violence by their partner or sexual violence from a non-partner at least once in their lifetime [1,2]. Gender-based violence against women is increasingly recognized as a major public health issue, including in Sustainable Development Goal 5 and many national policy strategies [3,4]. Such violence takes different forms, including domestic violence (DV) which refers to diverse acts of physical, sexual, and emotional actions that are committed by intimate partners or ex-partners to gain or maintain power or control, or cause harm. We followed the Jordan Population Family Health Survey (JPFHS) in using the term “domestic violence” throughout to mean domestic, spousal and intimate partner violence [5]. Domestic violence, “a pattern of behavior in any relationships used to gain or maintain power and control over an intimate partner” has been found to be associated with detrimental effects on health, including on reproductive health, physical and mental well-being, injury, chronic pain, drug and alcohol abuse, depression, and physical disability [6–9]. Marital control encapsulates controlling behaviors of the husband including jealousy/anger if the wife speaks to other men, frequent accusations, restrictive prohibition of meeting others, limitation of contact with the wife’s family, and constant tracking of whereabouts [5].

Domestic violence is a growing concern in the Middle East, including in Jordan [1,6]. Women in Jordan report high levels of all types of violence including emotional, sexual, and physical abuse [9,10]. The JPFHS suggest that, in 2007, more than 30% of ever-married women aged 15–49 reported experiencing physical violence since age 15. This prevalence increased to 34% in 2012 and then decreased to 21% in 2017–2018 [5]. Only 29% of this population of women reported having never experienced any controlling behaviors by their husbands and 15% state that their husbands do not allow them to meet their female friends [5]. Overall, these estimates are concerning, especially allowing for a likely under-reporting in surveys [11].

Heise’s integrated ecological framework of violence conceptualizes domestic violence as the result of risk factors at four levels: the macrosystem, exosystem, microsystem, and ontogenic system [7]. Societal risk factors in the macrosystem consist of masculinity linked to aggression, male perception of ownership of women, acceptance of interpersonal violence and physical chastisement, and rigid gender roles. At the exosystem level, risk factors include isolation of women and family, delinquent peer associations, and low socioeconomic status/unemployment, at the microsystem level; male dominance and control of wealth, alcohol use, and marital/verbal conflict, and at the personal history level; history of witnessing marital violence, past experience of abuse as a child, and an absent/rejecting father [7]. Economic factors can also affect domestic violence at the different levels: there is mixed evidence on whether an increase in men’s employment results in lower or higher incidence of domestic violence [12]. Meanwhile, an increase in women’s unemployment appears to increase domestic violence in low-and-middle-income countries (LMICs), possibly because women have decreased economic power and empowerment, increased stress, and less time outside of the home which can be contributing risk factors to domestic violence against women [13,14].

Applications of the ecological model to Jordan have identified several risk factors specific to the Jordan context [15]. Macrosystem factors include justifying violence, fearing family shame and social stigma of divorce, overarching religious traditions, and dominating male patriarchy and gender role inequalities. Exosystem factors include tolerance of abuse and hiding the violence, limited community resources, and barriers to services utilization [15]. Additional risk factors for domestic violence include lower levels of education, witnessing family violence, antisocial personality disorder, harmful masculine behaviors, and community norms that ascribe higher status to men [16,17]. Existing evidence in LMICs also suggests that educational attainment may be protective of violence, whereas alcohol use and being involved in a polygynous union are associated with higher risk of violence [5,18].

An important recent stressor in Jordan may be the large influx of refugees from the 2011 Syrian civil war into Jordanian host communities. By 2020, more than 6.7 million Syrians had been displaced across borders, mostly to neighboring countries such as Lebanon, Turkey, and Jordan [19] (S1A Fig). With a population of about 10 million, Jordan received some 660,000 Syrian peoples registered by the UNHCR as “refugees” by 2017, and had an estimated total of 1.3 million registered and unregistered Syrian refugees [20]. Jordanian governorates close to the Syrian border, such as Mafraq, Amman, and Irbid, received a high number of refugees in absolute terms (S1 Table).

In the context of the ecological model, this increase of refugees could affect the prevalence of violence in the Jordanian host community in multiple ways [7,15]. First, at the exosystem level, more competition in the formal and informal job market with the increase of refugees may exacerbate risk factors of domestic violence including unemployment and concentrations of poverty. Jordan’s labor market and economy were already fragile before 2011 and unemployment increased from 14.5 to 22.1 percent from 2011 to 2014 [21,22]. Refugees have limited ability to engage in Jordan’s formal sector work because of legal restrictions and administrative requirements, but they may accept lower pay and longer hours working in the informal sector [22,23]. This may have increased economic pressures and stress, especially for Jordanian households [24]. Furthermore, the effects of the Syrian refugee influx may be intensified by the intersection of risk factors by gender and employment, as mentioned previously [15].

Second, most Syrian refugees live in Jordanian communities rather than refugee camps. The influx thus increased the concentrations of people with past experiences of abuse and trauma which could impact the social and community norms and change the socio-demographic structure [25]. Jordan’s National Resilience Plan notes that refugee crises may have put pressure on coping mechanisms for Jordanian host communities and that social tensions have created an atmosphere of increased unrest and violence due to the heightened sense of insecurity in the population [24]. Also, the increased population may also strain the local communities’ access to and utilization of public health services [26].

Additionally, justification of violence may be associated with higher rates of domestic violence [7]. At the macrosystem level of the ecological model, this may occur because of existing social norms of male dominance in Jordanian society that justify violence and allow males to believe that DV is “common” or “normal” [15,27]. The 2017–18 JFPHS found that 46% of women and 69% of all men ages 15–49 justify wife beating under a specified circumstance. Justification of DV may become more common in communities that experience high rates of such violence, which could affect comparisons of reported DV. In particular, comparisons based on reported DV may be biased toward finding no differences. Such changes in reporting can lead to erroneous conclusions.

In this study, we empirically examined the effect of the sharp and large influx of Syrian refugees on domestic violence among Jordanian women in Jordanian host populations. We used data from the 2017–18 JPFHS to examine domestic violence and specific components of the overall construct, including physical, sexual, and emotional violence. We also examined effects on any physical or sexual violence, effects on employment (as a potential channel), as well as justifications for wife beating. We utilized regression-adjusted inverse propensity score weighting (IPWRA) to compare outcomes in the JPFHS clusters that are comparable on observable characteristics but differ in the share of Syrian women in the local population [26].

## Materials and methods

### Data

The 2017–18 JPFHS is a Demographic and Health Survey that collected nationwide data from October 2017- January 2018 [5]. The survey utilized a two-stage cluster design and separated each governorate into urban and rural regions. In the first stage, 970 clusters, or enumeration areas, were selected with a probability proportional to size and within each cluster, 20 households were randomly selected for interviews [26]. The target population was women ages 15–49 years old and the response rates for households and women of childbearing age are above 98%. The JPFHS collects information on a wide spectrum of maternal and child health indicators including physical violence, antenatal care visits, martial control, and early childhood development. The 2017–2018 JPFHS included a dedicated module for domestic violence that was administered to one randomly selected, ever-married woman in each household. For this study, we subset our data to the population of women that were currently in union and/or have a partner.

**Fig 1:**
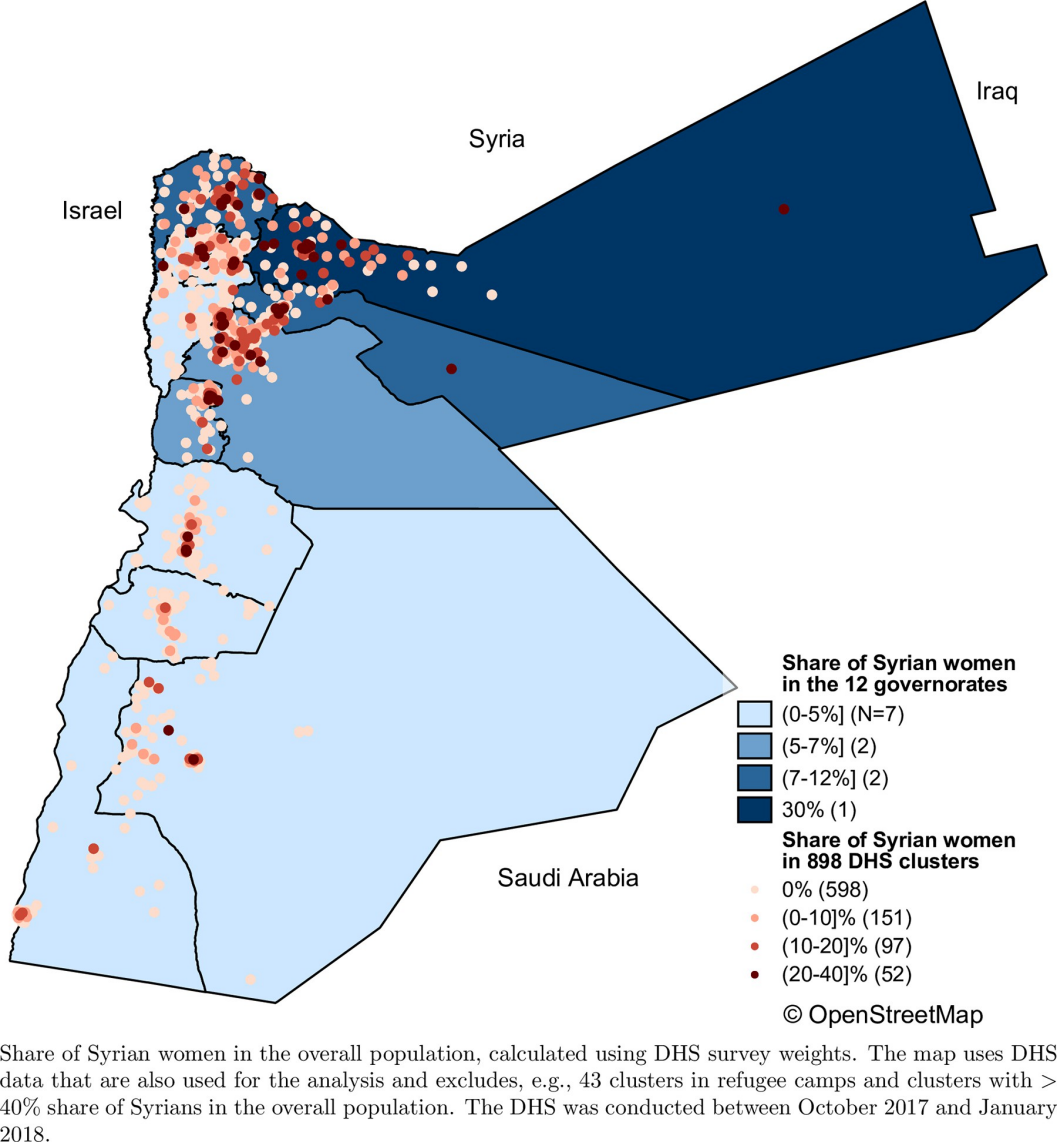
Distribution of Syrian women in Jordan. A) Share of Syrian women in the 12 governorates with darker colors representing a higher density B) Share of Syrian women in 898 DHS clusters with darker colored dots representing a higher density [28].

We imposed several restrictions to obtain our analysis sample (S4 Fig). First, excluded 43 clusters that the JPFHS specifically selected from Syrian refugee camps in the Mafraq and Zarqa governorates. Second, we focused on Jordanian nationals who are residents at the interview location (rather than visitors). Third, we excluded clusters with shares of Syrian women of more than 40% because of the small sample size and wide range in the share (Fig 1). We also S1B Fig showed the variation in the density of urban registered refugees across the remaining 898 PFHS clusters.

Next, we described the exposure and outcome measures, and the covariates. The question wording and corresponding coding of each variable are included in the S3 Table.

#### Exposure measures

We used a cluster-level measure of the density of Syrian women as our exposure. Specifically, we used sampling weights provided by the PFHS to calculate the percentage of Syrian women among all women in a cluster. Then, we created a categorical variable that indicates whether Syrian women in a cluster represent 0, 0^+^-10, 10–20 and 20–40 percent of all women eligible for a PHFS interview. The PFHS only records self-reported nationality but not refugee status or when a foreign-national respondent arrived in Jordan. We omitted from the share calculation those respondents who stated that they are Syrian nationals and “always” resided at the place of the interview.

#### Outcome measures

Our outcomes included two distinct measures of domestic violence, direct violence from a partner (including marital control) or from anyone and justification of violence, within Jordanian populations. Among women who were selected for the domestic violence survey module, we considered as an outcome: any physical, emotional, and sexual spousal violence, and partner’s employment status in the last 12 months before the 2017–18 JPFHS interview. We also assessed physical/sexual violence by anyone in the last 12 months, among all women. We used a binary measure for each of the outcomes indicating whether each form of physical, emotional, and/or sexual violence was experienced “often” or “sometimes” as opposed to “yes but not in the last 12 months” or “never”. Finally, we examined whether a husband displays behaviors of marital control, including being jealous or angry if she talks to other men or insisting on her whereabouts at all times.

We also examined women’s rationalization of domestic violence within the population of women who have a partner. There are seven subsections including justifying domestic violence: if the wife insults the husband, disobeys the husband, has a relationship with another, goes out without telling the husband, neglects the children, argues with the husband, or burns the food. We create a binary measure using the “yes” and “no” responses, omitting answers of “don’t know”. “Don’t know” responses constituted less than 1% of responses for all questions except “relations with another man” for which 2.1% of responses were “don’t know”. Finally, we also examined effects on employment of the female respondent and her partner, which may have been altered due to the exposure of the refugee influx.

#### Covariates

We considered covariates at the cluster, household and individual level that were found to be relevant in the existing literature for exposure or outcomes (see below for details on this distinction in the empirical model) [15,25]. At the cluster level, we adjusted for median wealth quintile among Jordanian households (as provided by the JPFHS), an indicator for whether the cluster is urban, and four indicator variables for the quartiles of the distance of the cluster to the Syrian border. The household-level covariates were use of internet in the last 12 months, as a proxy for socio-economic status and connectedness (binary variable of whether the respondent has used the internet in the last 12 months), household size (five or more members) and wealth quintile. The respondent-level covariates included binary variables of whether the respondent and husband had more than secondary education, whether the respondent first cohabitated at age 18 or older, and whether the husband is 10 years or more older than the respondent [29,30].

We estimated average treatment effects on the treated (ATET) using multi-level regression-adjusted inverse propensity score weighting (IPWRA) with standard errors clustered at the level of enumeration areas. IPRWA consists of an outcomes model that is a linear regression with weights derived from propensity scores that, in turn, are estimated in a logit treatment model. IPWRA is a “doubly-robust” estimator that is consistent as long as one of the models is correctly specified [31,32]. The lower panels of Table 1 listed the covariates included in the two constituent models. In particular, exposure was modeled using the cluster-level and household-level covariates listed above, while the outcomes model used household-level and respondent-level covariates.

**Table 1:** Summary statistics for the full analysis sample by treatment level (mean prevalence across clusters in percent and sample size).

	Percent of Syrian women in DHS enumeration area									
	0%		(0–10] %		(10–20] %		(20–40] %		Total	
	Mean	Count	Mean	Count	Mean	Count	Mean	Count	Mean	Count
**Dependent variables**										
*Any violence*										
Any physical violence	12.24	3,871	10.67	890	11.78	518	10.14	217	11.86	5,496
Any sexual violence	2.71	3,871	3.26	890	3.86	518	4.15	217	2.97	5,496
*Violence by partner*										
Partner any type of violence	17.44	3,871	16.97	890	17.95	518	16.13	217	17.36	5,496
Partner physical violence	10.54	3,871	8.88	890	9.85	518	8.29	217	10.12	5,496
Partner sexual violence	2.71	3,871	3.26	890	3.86	518	4.15	217	2.97	5,496
Partner emotional violence	13.48	3,871	13.6	890	14.48	518	12.44	217	13.56	5,496
*Marital control*										
Husband controlling behaviors*±*	14.29	3,871	13.71	890	13.51	518	17.51	217	14.25	5,496
*Beating wife is justified if she*.* *.* *.										
Insults husband	19.12	7,995	18.53	1,900	17.24	1,114	23.17	479	19.01	11,488
Disobeys husband	14.17	8,003	13.02	1,905	13.42	1,118	14.88	477	13.94	11,503
Has relations with another man	48.31	7,897	48.91	1,873	48.68	1,099	50.53	473	48.54	11,342
Goes out without telling husband	8.3	8,021	7.45	1,907	6.8	1,118	9.6	479	8.07	11,525
Neglects the children	6.87	8,017	7.24	1,907	7.87	1,118	9.75	482	7.15	11,524
Argues with husband	8.15	8,008	6.6	1,909	6.17	1,119	5.85	479	7.61	11,515
Burns the food	3.34	8,024	2.35	1,912	3.22	1,118	0.83	480	3.06	11,534
*Employment*										
Respondent works	14.18	8,052	17.56	1,919	15.08	1,121	13.64	484	14.81	11,576
Husband works	73.67	8,052	74.78	1,919	77.7	1,121	76.03	484	74.34	11,576
**Covariates**										
*Outcomes model (respondent level)*										
More than secondary education	35.22	8,052	39.81	1,919	35.86	1,121	37.19	484	36.13	11,576
First cohabitated aged 18 or older	81.52	8,052	81.66	1,919	81.53	1,121	82.64	484	81.59	11,576
Husband has more than secondary education	23.52	8,052	25.59	1,919	26.23	1,121	28.31	484	24.33	11,576
Husband is 10 or more years older	16.59	7,564	17.97	1,797	18.56	1,045	17.41	448	17.04	10,854
*Treatment and outcomes models (household level)*										
Used internet in last 12 months	72.12	8,052	78.01	1,919	75.91	1,121	78.93	484	73.75	11,576
Five or more household members	63.74	8,052	65.82	1,919	65.48	1,121	66.12	484	64.35	11,576
Wealth quintile	2.77	8,052	2.87	1,919	2.86	1,121	2.81	484	2.8	11,576
*Treatment model (cluster level)*										
Median wealth quintile*†*	2.72	8,052	2.83	1,919	2.87	1,121	2.77	484	2.76	11,576
Urban	0.72	8,052	0.84	1,919	0.92	1,121	0.93	484	0.77	11,576
Distance to Syrian border in km*‡*	122.42	8,052	80.5	1,919	71.44	1,121	51.69	484	107.58	11,576
**Other:** *Residency at interview location*										
Resident since 2010 or earlier	95.16	8,052	92.23	1,917	92.51	1,121	91.32	484	94.25	11,574
Resident since 2016 or earlier	0.99	8,052	0.99	1,917	0.99	1,121	0.99	484	0.99	11,574

*±* Husband displays 3 or more of 5 controlling behaviors: Jealousy, accuse of being unfaithful, does not permit to meet friends, tries to limit contact to her family, insists on knowing whereabouts at all times.

*†* Median wealth quintile for Jordanian households.

*‡* The treatment model of the IPRWA analysis uses quartiles of distance.

**Fig 2:**
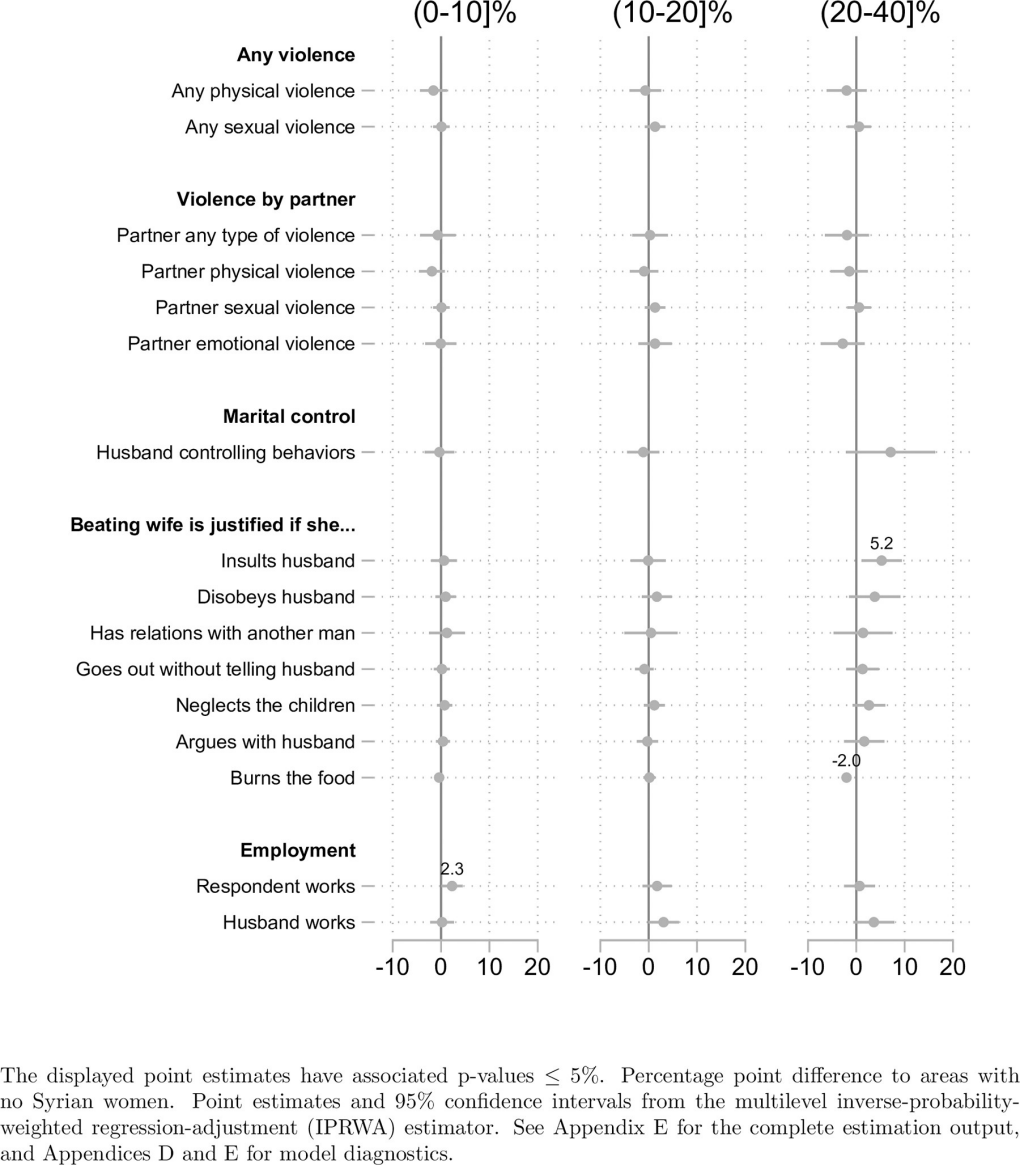
Treatment effects by share of Syrian women in a DHS enumeration area (percentage points, previous 12 months). Estimates of treatment effects with p-values less than 5% from the IPRWA estimator are labeled with the exact percentage point difference value compared to areas with no Syrian women.

We did not formally account for multiple comparisons but constructed indices from the multiple violence measures, following Kling and colleagues [33]. We used a significance level of 5% to present our main findings (Fig 2). We used Stata MP-17 Statistical software for all analyses [31]; the analysis code is available at [the repository will be included here upon publication; please see attached code for any review] while the data can be obtained from the Demographic and Health Survey website upon registration. This study was approved by the institutional review boards of the Harvard T.H. Chan School of Public Health.

## Results

Table 1 shows descriptive statistics for the analytical sample and the flow chart in S4 Fig displays the flow of selected and excluded clusters/study participants for the final analytical sample of 11576 women. The total sample sizes of women vary by dependent variable and range from about 5,500 for the violence measures to 11,500 for the employment and rationalization measures. The reason for this discrepancy is that the violence module was administered to a random subset of respondents. The bottom panel of the table shows that JPFHS clusters with a higher share of Syrian women are more likely to be urban and closer to the Syrian border. Almost all respondents have lived in the enumeration area since at least 2016, that is, prior to the beginning of the recall period.

Overall in our sample, experiences of violence are not rare. About 12 percent of respondents reported having experienced any physical violence in the last 12 months, and 17 percent reported experiences of any partner violence in that period. Partners are the majority of perpetrators of DV events. Reports of “any” physical violence are highly correlated with physical violence by a partner, and women who report any sexual violence also report sexual violence by a partner. About 15 percent of respondents report working, while almost three-quarters reported that their husband works. Almost half of the respondents agreed that a husband beating his wife is justified if she has extra-marital relations; 19 percent justified a husband beating his wife insults the husband.

Fig 2 summarizes the results from the empirical analysis by displaying the coefficient estimates and 95% confidence intervals, as well as the value of the point estimate if it is statistically significant at p≤5%. S1 Dataset exhibits the complete estimation results with exact p-values as well. S3A–S3D Fig contains model diagnostics, including overlap graphs, standardized differences, and variance ratios, and full estimation results underlying Fig 2 are included in S1 Dataset. We did not find any statistically significant impacts on reported violence, overall and for different types of partner violence. We also did not find substantial effects on women or husband’s employment as a potential risk factor for DV; respondent employment increased by 2.3 percentage points in areas with 0–10 percent Syrian women, relative to areas with no Syrian women. We did not detect statistically significant differences across areas with no or different shares of Syrian women on husband’s martial control behaviors. Finally, we did not detect systematic differences in the justification for violence across communities: in enumeration areas with 20–40 percent Syrian women, Jordanian women are more likely to justify wife beating if the wife insults the husband (5.2 percentage points) but slightly less likely to justify beating for burning food (negative 2 percentage points). We obtained a similar pattern of results in the robustness check that limits the sample to respondents who resided at the interview location since 2010 (S2 Fig).

We conducted ex-post power calculations to obtain minimum detectable effect sizes (MDE) based on the actual sample sizes for each dependent variable and empirical parameters from the control areas (S2 Table). The estimated MDEs for the violence outcomes range between 2 (sexual violence) and 4.2 (any partner violence) percentage points. While there are important caveats with this kind of calculation, we note that these MDEs are large relative to the overall prevalence of, e.g., sexual violence (3 percent) or any partner violence (17 percent) as reported in Table 1. This suggests that our sample may be too small to statistically detect differences in violence that are substantively meaningful and important.

## Discussion

Our study utilized a multilevel IPRWA estimation approach to determine the treatment effects of the influx of Syrian refugees on domestic violence among Jordanian host populations. We found no statistically significant difference between domestic violence experienced by Jordanian women between areas with low or high density of Syrian women, our proxy for the presence of refugees. We also did not find evidence for changes in employment as a potential pathway for violence, nor changes in justification for wife beating and marital control. However, our ex-post power calculations indicated that there could be meaningful effects on violence that we cannot statistically detect with the available sample. In addition, although we did not find evidence for increases in DV or marital control, these issues remain pressing concerns in Jordan given the high prevalence (21%) of these events as measured by the JPFHS.

We hypothesized that the influx of refugees might impact the levels of domestic violence among Jordanian host communities through an economic channel in which unemployment changes as many Syrian refugees enter the formal and informal labor market in Jordan, at the exosystem level of the ecological model of violence However, our study revealed that the difference in density of refugees in enumeration areas does not systematically change employment among respondents nor among their husbands. Rather, we noticed that areas with a low relative exposure of Syrian women (0–10%) had higher rates of employment among respondents (2.3 percentage points) compared to areas with zero exposure (0% density of Syrian women). These findings did not support the existence of the employment channel.

Additionally, according to the ecological model, at the macrosystem level, rationalization of violence could have impacted reporting and resulted in a null finding. However, our results revealed no evidence of differential (increased) rationalization among enumeration areas with a relatively higher density or exposure of refugees. In either case, our findings also did not suggest that differential reporting drove the lack of observed impacts. It is possible that rationalization was not impacted or that justification was already so prevalent that the additional stressors only had a small (not detectable) effect.

Another explanation for these findings may be that efforts by the government and non-governmental organizations (NGOs) were effective at mitigating the impacts of refugees on host communities. For example, Jordan’s National Employment Strategy (2011–2020) aimed to address employment challenges in Jordan, focusing on the inclusion of women and youth in the workforce [22]. Especially for targeted poor groups of vulnerable Jordanians, the National Aid Fund and Zakart Fund both provide cash assistance to cover basic needs and supplement the increasing cost of living [22]. The government has also put in place a specific effort to curb the incidence of violence in recent years from the Family and Protection Department (FPD) through protection services and social services to childhood and women. The Ministry of Social Development (MoSD) estimated an increase of 8 million JOD (11.3 million USD) in expenses due to the Syrian refugee crisis [22,34]. The government reported that the FPD has seen a steady increase in reporting of violence but that this may not necessarily be due to an increase in incidence of violence but rather, an increase in awareness campaigns, data collection, improved monitoring mechanisms, and wider geographical reach [22].

Similarly, policies and interventions to protect women from domestic violence and to offer recourse to DV victims may have helped avoid an increase in DV. In Jordan, this includes the Law Regarding Protection from Domestic Violence that was promulgated in 2008 and enhanced protections, such as maintaining confidentiality for victims and ensuring protectionary measure for informants [35]. Jordan also ratified international conventions (including the Convention on the Elimination of All Forms of Discrimination against Women, CEDAW), participated in the global movement combating violence against women, and in 2013, approved the National Strategy for Jordanian Women, targeting women’s rights and empowerment [34,35]. However, effective protection and prevention has remained limited in practice [34]. In this regard, support for Jordan’s National Commission for Women (JNCW) in implementing CEDAW could be helpful. Additional efforts include strengthening education and vocational training, and generally providing more labor market opportunities for women which may be protective.

Our analysis had several limitations. First, our empirical approach balanced observable factors across the exposed and unexposed groups but was at risk of unobservable characteristics that may differentially influence the treatment effect and lead to a positive or negative bias in our estimates. Second, Jordanian households could have relocated due to the influx of Syrian refugees. Although Table 1 shows that almost all respondents lived at the interview location since 2016 (the year when our 12 months recall starts for respondents interviewed in late 2017), we conduct a robustness check in which we focused on respondents who lived at the place of interview since 2010 and found that our findings still held. Third, our exposure variable of the density of Syrian women was calculated in 2017–2018 and fixed but there is a possibility that Syrian households may have moved over time of the recall period, which could lead to discrepancies between the actual and measured exposure. However, aggregate count and distribution of Syrian refugees across governorates were quite stable between 2014–2017 (S1 Table). Fourth, differential misreporting of DV in areas with a higher or lower share of Syrian women may affect our results, e.g., if Jordanian women in high-exposure areas have higher stigma they may be less likely to report on violence. Additionally, if institutions in communities with large influxes of refugees are overwhelmed, women have increased barriers to accessing the police, clinics, or organizations to report cases. We found two statistically significant effects in variables on the justification of wife beating, the two estimates are in opposite direction and there are no detectable differences in the remaining five variables on justification. This was consistent with no differential misreporting, although we cannot fully rule out this risk to our estimates. Finally, because of sample size limitations we could not explore whether there may be additional heterogeneity in the impact, e.g., due to regional and local social or cultural factors. Future research could further explore this possibility.

## Conclusion

Our study did not detect significant impacts of the presence of Syrian women on domestic violence among Jordanian women in the same communities. The influx of refugees was not associated with a change in domestic violence or marital control. However, we cannot rule out that there were substantively meaningful effects, beneficial and/or harmful, on violence that are statistically not detectable with our study sample. Further research, including qualitative studies, are needed to further investigate this major public health issue. Similarly, our findings point to the importance of enacting policies and interventions that reduce violence against women and increase support for Jordanian host communities in coping with the large influx of refugees.

## Supporting information

S1 Fig **A. Changes in the number of Syrian refugees in Jordan (1,000).** Monthly average changes from January 2012 to January 2019. **B. Percent of Syrian women in a community by distance to the Syrian border.** A) All 898 clusters (DHS enumeration areas) B) Clusters with a positive share of Syrian women.
(TIF)

S2 Fig Robustness check- Treatment effects for the sample that resided at interview location since 2010 or earlier (percentage points, previous 12 months). Each percentage point difference to areas with no Syrian women. Estimates of treatment effects with p-values less than 5% from the IPRWA estimator are displayed with the exact value.
(TIF)

S3 Fig A. Detailed output for the Inverse Probability Weighted Regression Adjustment (IPWRA) Model of Any violence. A) Black line: Treat = Share 0% (no overlap) B) Green line: Treat = Share (0–10]% C) Blue line: Treat = Share (10–20]% D) Brown line: Treat = Share (20–40]%. **B. Detailed output for the IPWRA Model of Partner violence.** A) Black line: Treat = Share 0% (no overlap) B) Green line: Treat = Share (0–10]% C) Blue line: Treat = Share (10–20]% D) Brown line: Treat = Share (20–40]%. **C. Detailed output for the IPWRA Model of Overlap for employment.** A) Black line: Treat = Share 0% (no overlap) B) Green line: Treat = Share (0–10]% C) Blue line: Treat = Share (10–20]% D) Brown line: Treat = Share (20–40]%. **D. Detailed output for the IPWRA Model of Overlap for justification.** A) Black line: Treat = Share 0% (no overlap) B) Green line: Treat = Share (0–10]% C) Blue line: Treat = Share (10–20]% D) Brown line: Treat = Share (20–40]%.
(TIF)

S4 Fig Flow chart of the analysis sample. The flow of selected and excluded clusters and study participants from the JPFHS data.
(TIF)

S1 Table Distribution of urban registered refugees across Jordanian governorates. The absolute counts and percentages of registered refugees in the 12 governorates from May 2014 to May 2017.
(TIF)

S2 Table Minimum detectable effect size (MDE). Ex-post calculations conducted to estimate MDE for a two-sample proportions test in a cluster randomized design (power = 0.80, alpha = 0.05).
(TIF)

S3 Table Questionnaire for all exposure, outcome, and covariate variables. Questions asked for the exposure variable, each outcome variable, and all covariate variables.
(TIF)

S1 Dataset Estimation results dataset. The full estimation results data in a spreadsheet csv file for the detailed results and power values.
(CSV)
