# Supplementary figures and images for: Impact of the influx of Syrian refugees on domestic violence against Jordanian women: Evidence from the 2017–18 Jordan Population and Family Health Survey
Source: PLoS One. 2023 Nov 8;18(11):e0288144. doi: 10.1371/journal.pone.0288144 (PMC10631676; doi:10.1371/journal.pone.0288144)

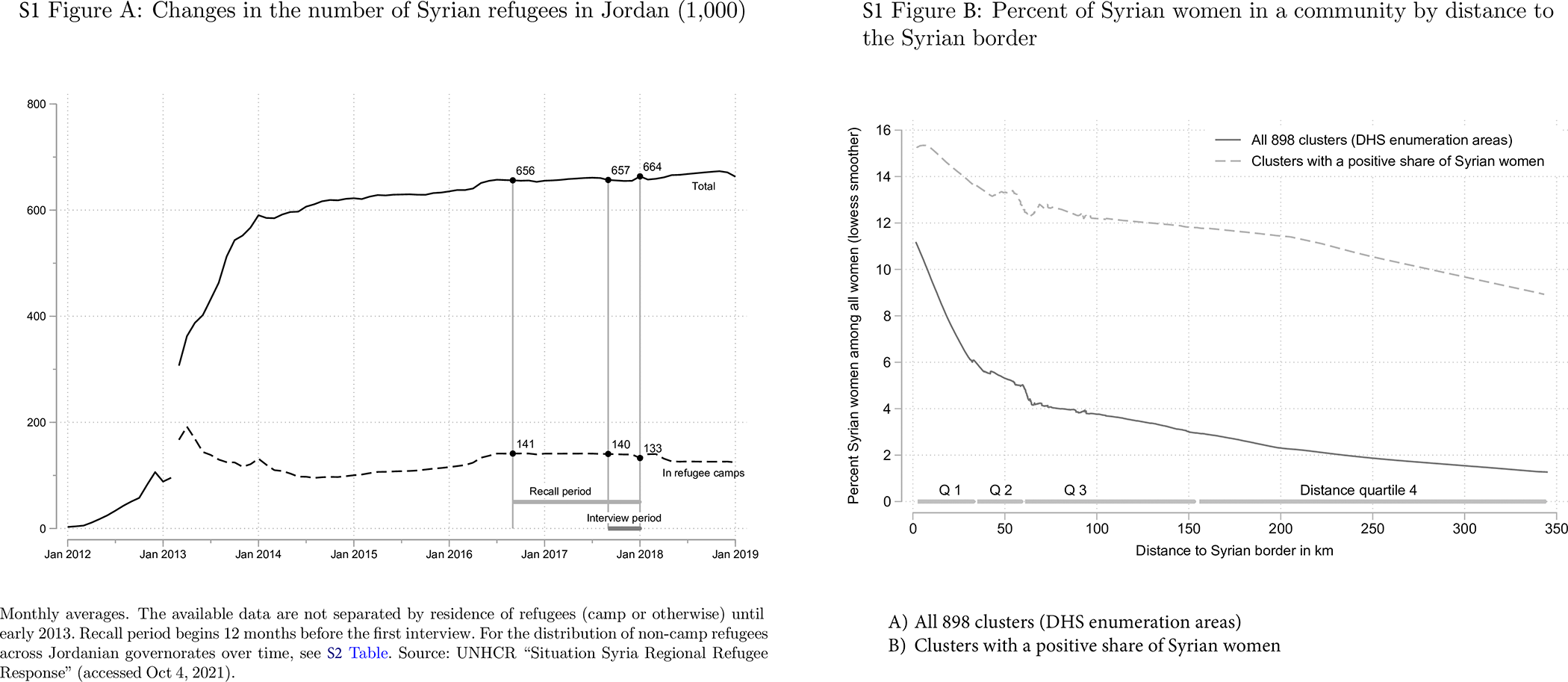

Supplement: S1 Fig — A. Changes in the number of Syrian refugees in Jordan (1,000). Monthly average changes from January 2012 to January 2019. B. Percent of Syrian women in a community by distance to the Syrian border. A) All 898 clusters (DHS enumeration areas) B) Clusters with a positive share of Syrian women. (TIF) [file pone.0288144.s001.tif]

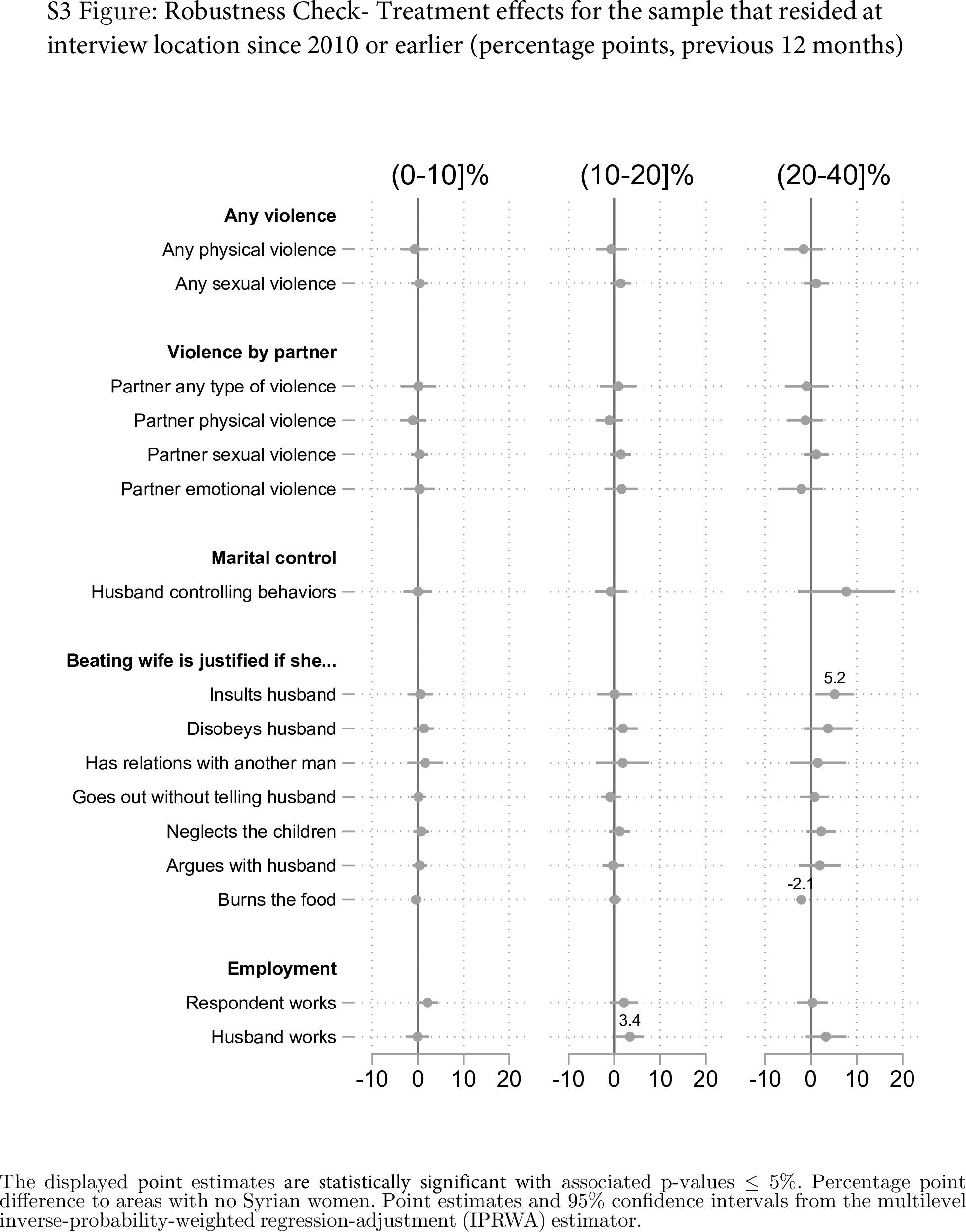

Supplement: S2 Fig — Each percentage point difference to areas with no Syrian women. Estimates of treatment effects with p-values less than 5% from the IPRWA estimator are displayed with the exact value. (TIF) [file pone.0288144.s002.tif]

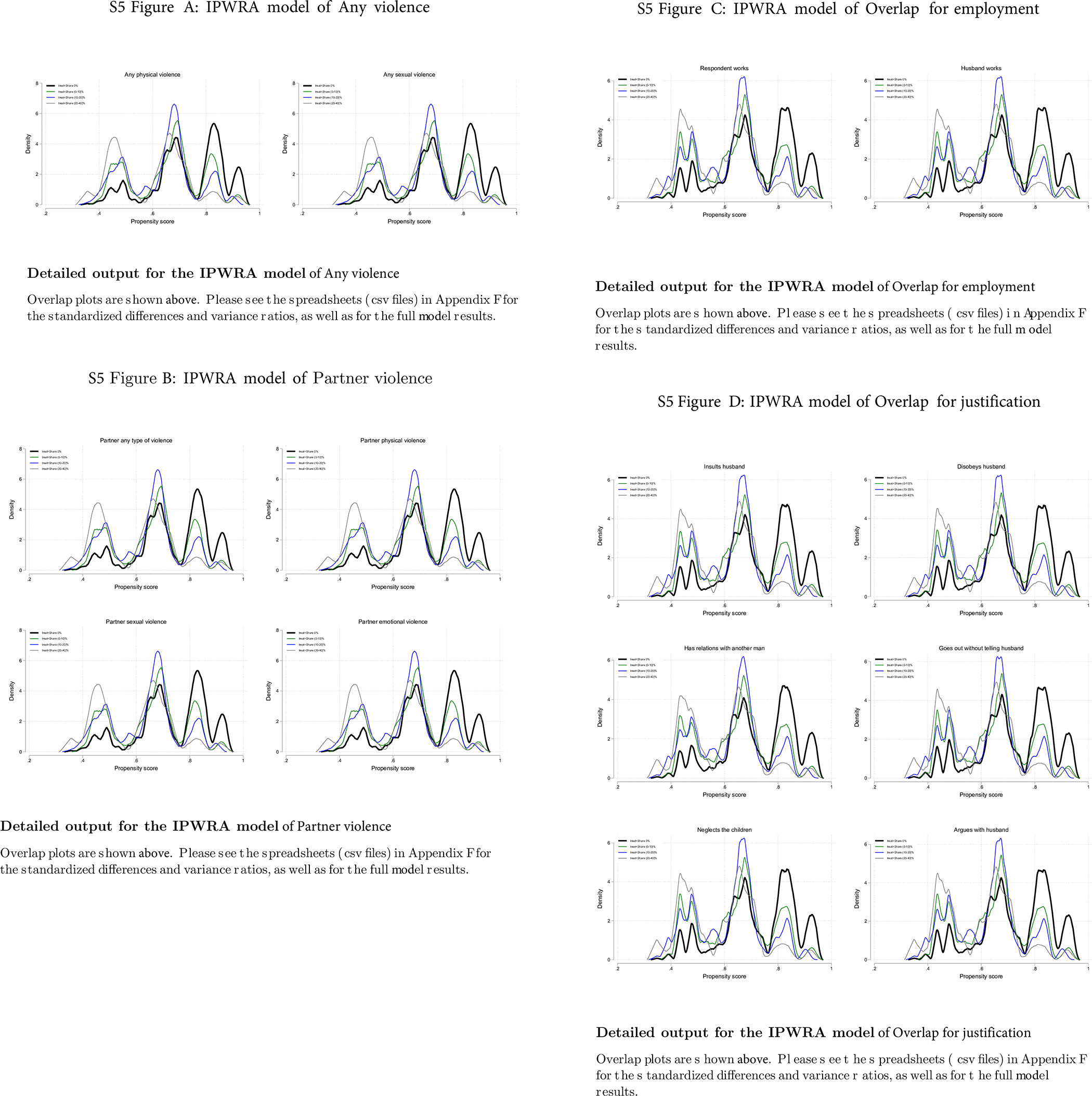

Supplement: S3 Fig — A) Black line: Treat = Share 0% (no overlap) B) Green line: Treat = Share (0–10]% C) Blue line: Treat = Share (10–20]% D) Brown line: Treat = Share (20–40]%. B. Detailed output for the IPWRA Model of Partner violence. A) Black line: Treat = Share 0% (no overlap) B) Green line: Treat = Share (0–10]% C) Blue line: Treat = Share (10–20]% D) Brown line: Treat = Share (20–40]%. C. Detailed output for the IPWRA Model of Overlap for employment. A) Black line: Treat = Share 0% (no overlap) B) Green line: Treat = Share (0–10]% C) Blue line: Treat = Share (10–20]% D) Brown line: Treat = Share (20–40]%. D. Detailed output for the IPWRA Model of Overlap for justification. A) Black line: Treat = Share 0% (no overlap) B) Green line: Treat = Share (0–10]% C) Blue line: Treat = Share (10–20]% D) Brown line: Treat = Share (20–40]%. (TIF) [file pone.0288144.s003.tif]

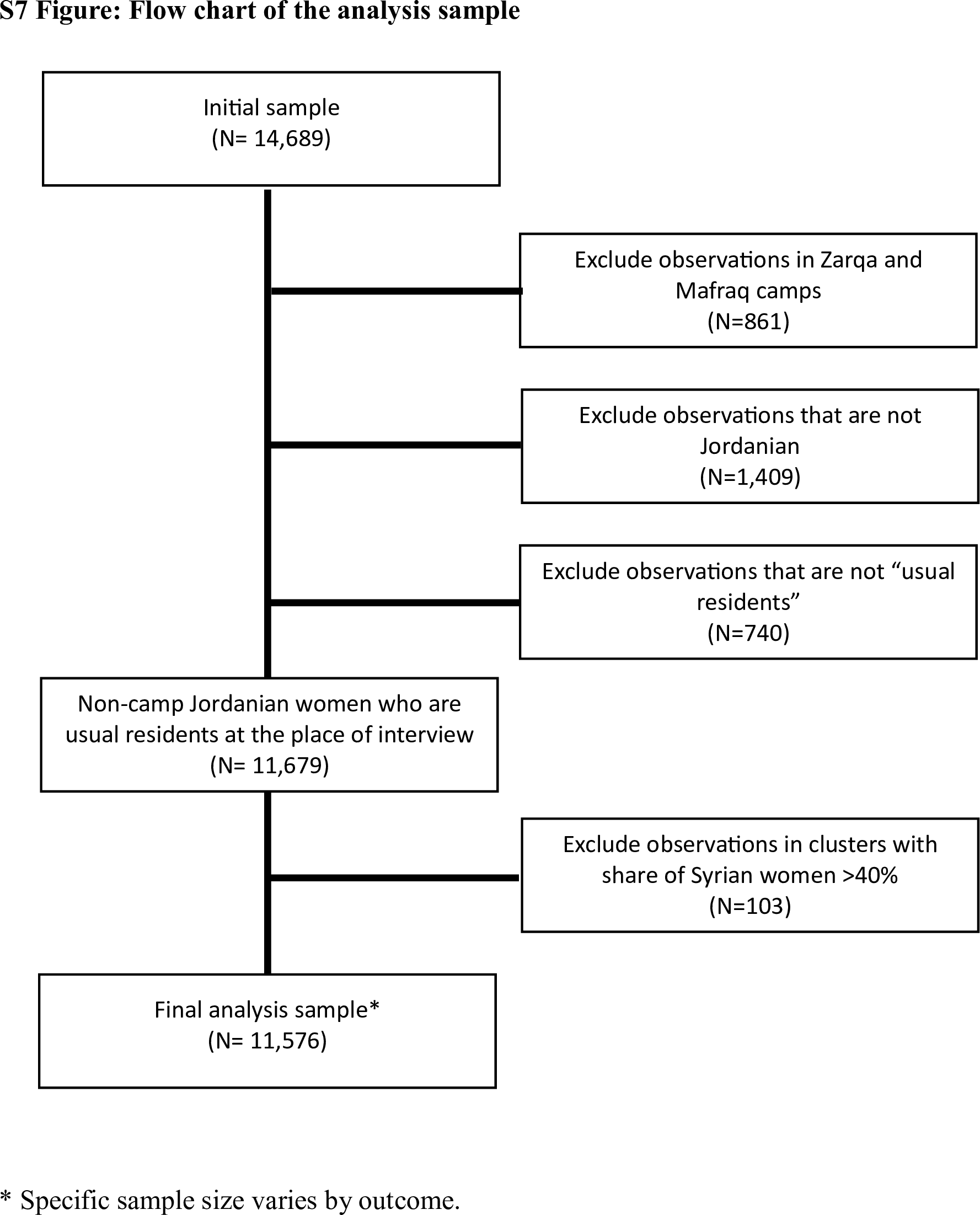

Supplement: S4 Fig — The flow of selected and excluded clusters and study participants from the JPFHS data. (TIF) [file pone.0288144.s004.tif]

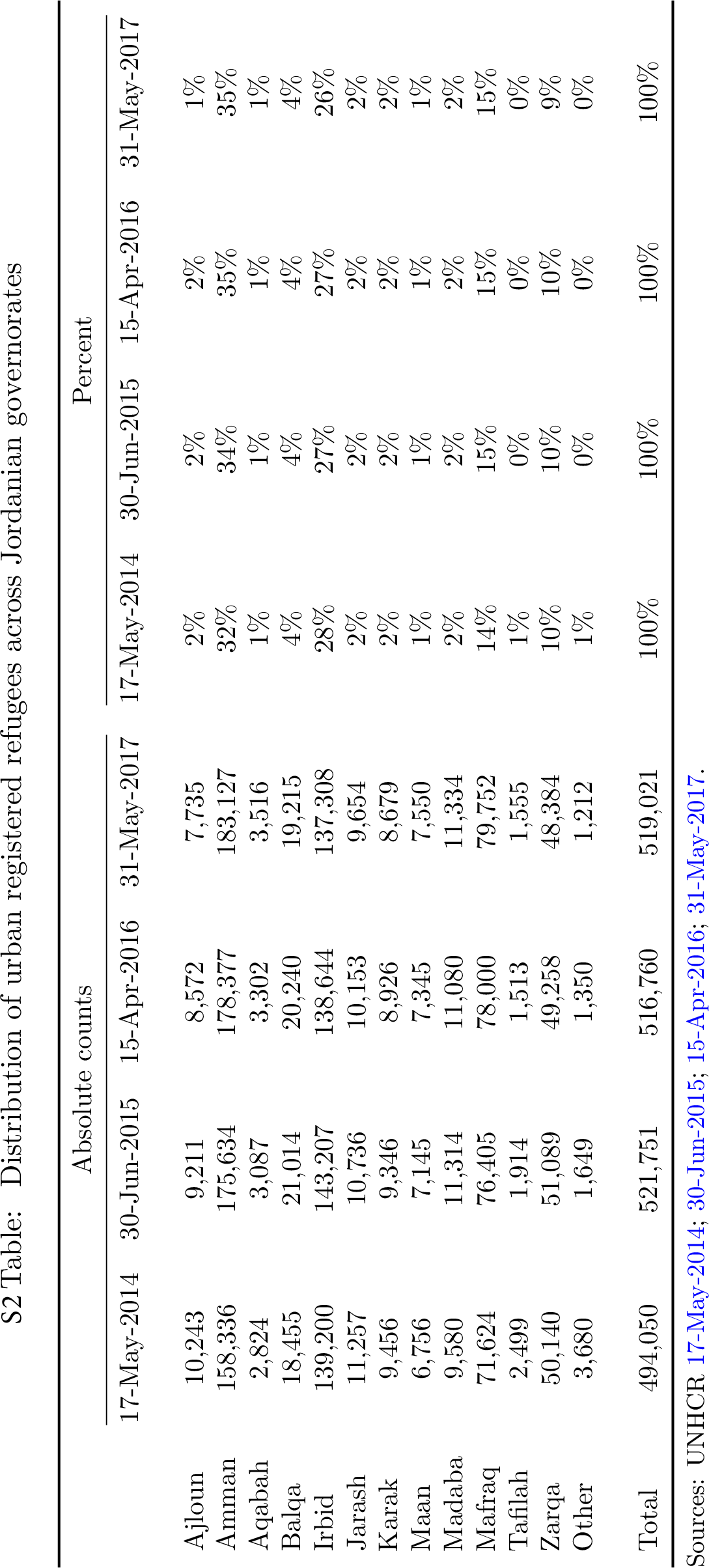

Supplement: S1 Table — The absolute counts and percentages of registered refugees in the 12 governorates from May 2014 to May 2017. (TIF) [file pone.0288144.s005.tif]

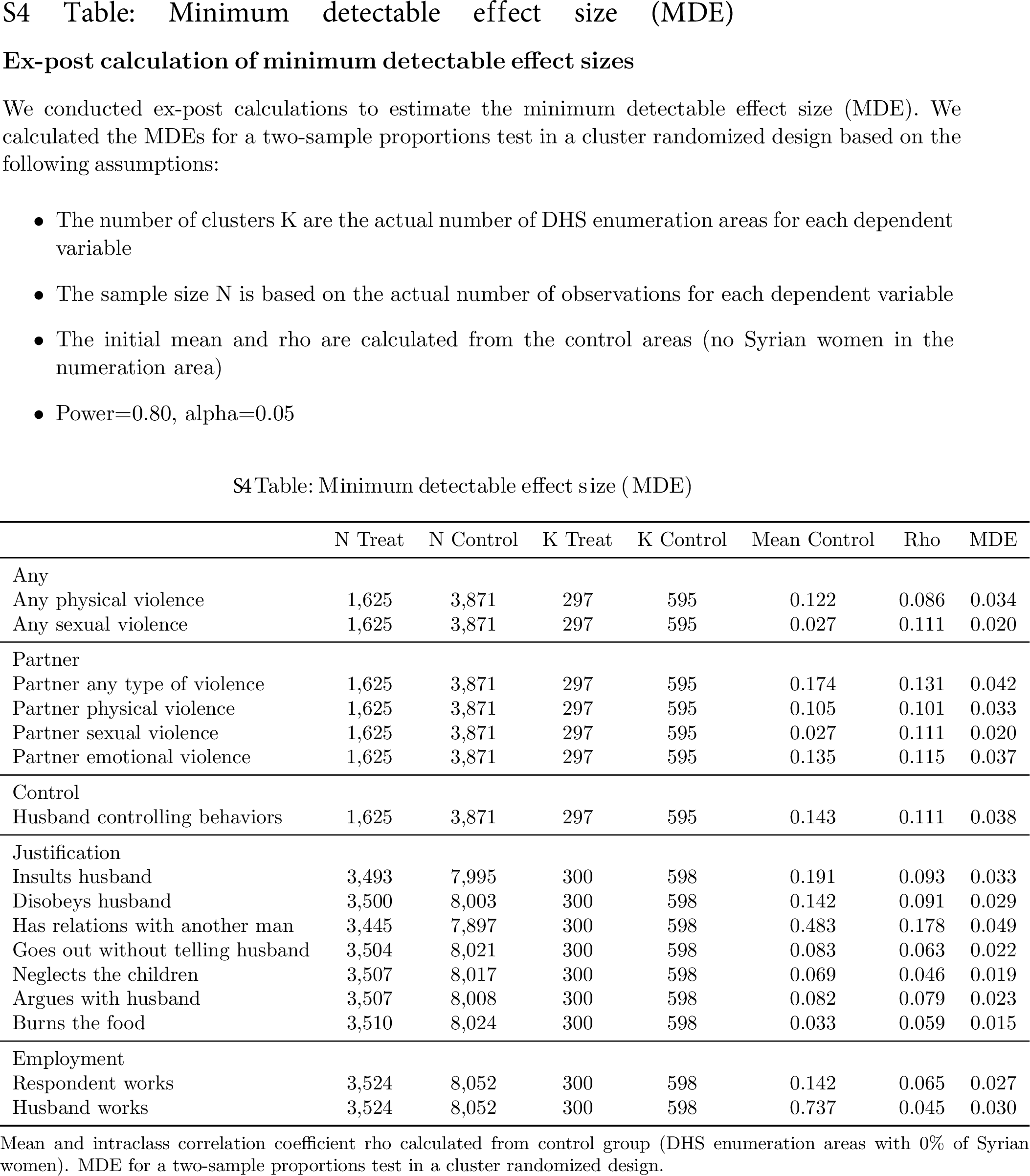

Supplement: S2 Table — Ex-post calculations conducted to estimate MDE for a two-sample proportions test in a cluster randomized design (power = 0.80, alpha = 0.05). (TIF) [file pone.0288144.s006.tif]

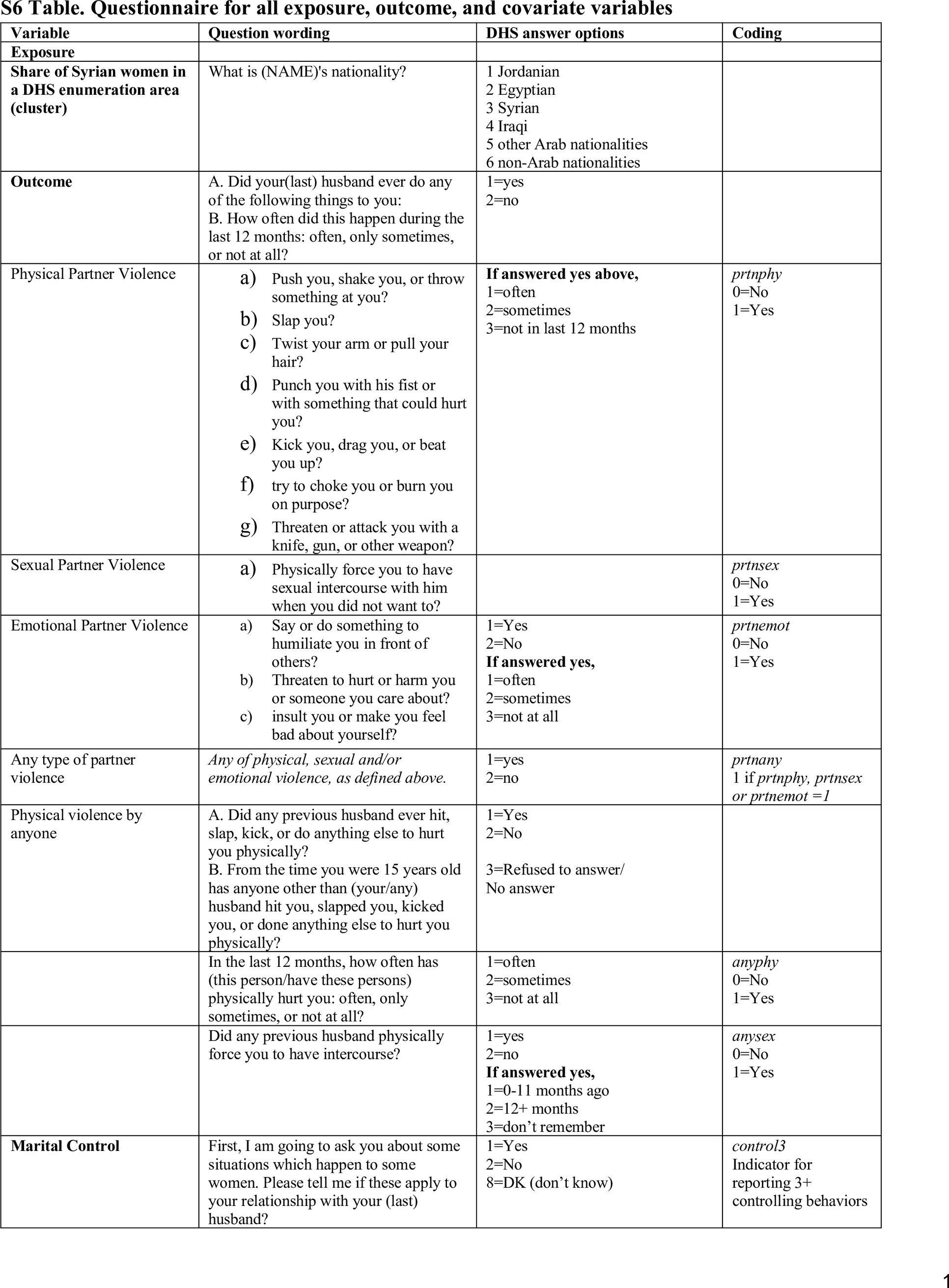

Supplement: S3 Table — Questions asked for the exposure variable, each outcome variable, and all covariate variables. (TIF) [file pone.0288144.s007.tif]
